# SARS-CoV-2 Recombinants: Genomic Comparison between XBF and Its Parental Lineages

**DOI:** 10.3390/microorganisms11071824

**Published:** 2023-07-17

**Authors:** Fabio Scarpa, Chiara Locci, Ilenia Azzena, Marco Casu, Pier Luigi Fiori, Alessandra Ciccozzi, Marta Giovanetti, Miriana Quaranta, Giancarlo Ceccarelli, Stefano Pascarella, Massimo Ciccozzi, Daria Sanna

**Affiliations:** 1 Department of Biomedical Sciences, University of Sassari, 07100 Sassari, Italy; c.locci3@phd.uniss.it (C.L.); iazzena@uniss.it (I.A.); fioripl@uniss.it (P.L.F.); darsanna@uniss.it (D.S.); 2 Department of Veterinary Medicine, University of Sassari, 07100 Sassari, Italy; marcasu@uniss.it; 3 Azienza Ospedaliera Universitaria (AOU) Sassari, 07100 Sassari, Italy; 4 Unit of Medical Statistics and Molecular Epidemiology, University Campus Bio-Medico of Rome, 00128 Rome, Italy; ale_ciccozzi97@icloud.com (A.C.); m.ciccozzi@unicampus.it (M.C.); 5 Sciences and Technologies for Sustainable Development and One Health, University of Campus Bio-Medico of Rome, 00128 Rome, Italy; giovanetti.marta@gmail.com; 6 Rene Rachou, Fundação Oswaldo Cruz, Belo Horizonte 30190-009, Minas Gerais, Brazil; 7 Department of Biochemical Sciences “A. Rossi Fanelli”, Sapienza Università di Roma, 00185 Rome, Italy; quaranta.1952503@studenti.uniroma1.it (M.Q.); stefano.pascarella@uniroma1.it (S.P.); 8 Department of Public Health and Infectious Diseases, University Hospital Policlinico Umberto I, Sapienza University of Rome, 00161 Rome, Italy; giancarlo.ceccarelli@uniroma1.it; 9 Campus Bio-Medico, Fondazione Policlinico Universitario, 00128 Rome, Italy

**Keywords:** coronavirus, SARS-CoV-2, genetics, genome diversity, epidemiology, recombination event, electrostatic surface potential, spike mutations, interaction energy

## Abstract

Recombination events are very common and represent one of the primary drivers of RNA virus evolution. The XBF SARS-CoV-2 lineage is one of the most recently generated recombinants during the COVID-19 pandemic. It is a recombinant of BA.5.2.3 and BA.2.75.3, both descendants of lineages that caused many concerns (BA.5 and BA.2.75, respectively). Here, we performed a genomic survey focused on comparing the recombinant XBF with its parental lineages to provide a comprehensive assessment of the evolutionary potential, epidemiological trajectory, and potential risks. Genetic analyses indicated that although XBF initially showed the typical expansion depicted by a steep curve, causing several concerns, currently there is no indication of significant expansion potential or a contagion rate surpassing that of other currently active or previously prevalent lineages. BSP indicated that the peak has been reached around 19 October 2022 and then the genetic variability suffered slight oscillations until early 5 March 2023 when the population size reduced for the last time starting its last plateau that is still lasting. Structural analyses confirmed its reduced potential, also indicating that properties of NTDs and RBDs of XBF and its parental lineages present no significant difference. Of course, cautionary measures must still be taken and genome-based monitoring remains the best tool for detecting any important changes in viral genome composition.

## 1. Introduction

Over the past years the world has experienced the COVID-19 pandemic caused by the SARS-CoV-2 virus, first detected in December 2019 during a pneumonia outbreak in Wuhan, China [1]. Following the initial cases, it rapidly became a worldwide issue [2], and on 11 March 2020, with a cumulative total of 149,295 confirmed cases, the World Health Organization (WHO) declared COVID-19 a pandemic [3]. As of 21 May 2013, a total of over 766 million confirmed cases and more than 6.9 million deaths have been reported globally by the WHO [4]. Despite the recent declaration by the WHO that declared the end of pandemic emergence, cautionary measures must still be taken regarding COVID-19 since SARS-CoV-2 continues to be present among the world population, is evolving, and infections caused by this virus are likely to persist as a problem in most countries. The WHO has emphasized the importance of not becoming complacent, as the SARS-CoV-2 virus is still evolving and circulating across the global population. Consequently, infections caused by this virus are expected to persist as a significant issue in many countries [5]. SARS-CoV-2 is a single-stranded RNA virus with a positive-sense genome and a high error rate in RNA replication, which leads to mutations affecting its susceptibility to neutralizing antibodies raised by infection and/or vaccination, as well as its transmissibility [6].

Consequently, during the pandemic, SARS-CoV-2 has undergone a high number of mutations over time, resulting in multiple lineages and sub-lineages with varying expansion capabilities [7]. Recombination events may also play a significant role in the generation of new viral variants. Indeed, the primary driver of RNA virus evolution is usually recombination [8], along with re-assortment (which is specifically involved in RNA viruses with segmented genomes like the influenza virus [9]). Naturally, the occurrence of recombination between different lineages necessitates the simultaneous presence and infection of viruses within the same host [10]. As for all new variants, the occurrence of new recombinants among newly discovered variants needs to be monitored through constant surveillance [11]. One of the most recent recombinants of SARS-CoV-2 is the XBB lineage [11,12]. The XBB lineage is a recombinant of two lineages, BJ.1 and BM.1.1.1, both belonging to the BA.2 lineage, with additional mutations within the spike protein and other viral genes [13].

The XBB recombinant has raised many concerns between late 2022 and early 2023. In particular, both XBB and its first descendant XBB.1 have shown concerning evidence of their ability to evade immunity provided by vaccination or prior infection, primarily due to mutations or loss of B cell epitopes [14]. However, individuals who are fully vaccinated still maintain protection against hospitalization and death due to the preserved antiviral activity of T cells targeting conserved T epitopes on the Spike protein [15]. Importantly, a comprehensive genomic analysis that combined multiple disciplines demonstrated that both XBB and XBB.1 lineages do not exhibit significant expansion capabilities or a higher contagion rate compared to other currently circulating lineages [11]. On the contrary, they have displayed a long period of flattened genetic variability [11], which indicates a low potential for epidemiological threat in terms of spread.

The last recombinant lineage generated by a recombination event is represented by SARS-CoV-2 XBF. Indeed, the XBF lineage is a recombinant of BA.5.2.3 and BA.2.75.3, both descendants of lineages that caused many concerns (BA.5 and BA.2.75, respectively) [16]. It presents three Spike mutations in addition to those typical of its progenitor lineages (R346T, F486P, and F490S) and one in the ORF1a gene (V1025A). Moreover, it shares several spike mutations with the parental lineage BA.2.75.3 (K147E, W152R, F157L, I210V, G257S, G339H, G446S, N460K) [17]. For details on the characterizing XBF mutations are reported in Supplementary Table S1.

As for all variants, overall, constant surveillance of the lineage’s genome, along with immunological and clinical monitoring, is crucial to identify and assess any mutations that could impact virus transmissibility, host immune responses, and pathogenicity. In such a context, we conducted a genomic survey focusing on genetic variability/phylodynamics, and structural analyses to provide a comprehensive assessment of the evolutionary potential, epidemiological trajectory, and potential risks of XBF and its descendant. In particular, the recombinant XBF has been compared with its parental lineages (BA.5.2.3 and BA.2.75.3), to identify any novel biological characteristics that could potentially contribute to its rapid spread and potential outcompeting of the original lineages.

## 2. Materials and Methods

### 2.1. Phylodynamics Analyses

In order to locate the SARS-CoV-2 XBF lineage from an evolutionary perspective, a preliminary phylogenomic analysis was conducted on the Omicron variants. This investigation was conducted by means of global data over the past six months using the nextstrain/ncov (https://github.com/nextstrain/ncov, accessed on 4 May 2023) tool (available at https://gisaid.org/phylodynamics/global/nextstrain/, accessed on 4 May 2023), including all genomes belonging to the GISAID Clade 21M (Omicron) (2350 of 2827 genomes sampled between November 2021 and May 2023). The analysis aimed to provide a comprehensive understanding of the evolutionary trajectory of the SARS-CoV-2 Omicron lineages.

After the first phylogenomic assessment, in order to conduct a genetic comparison between XBF and its parental lineages, three subsets were built: BA.5.2.3 (*n* = 384), BA.2.75.3 (*n* = 201), and XBF (*n* = 1867), and the genetic analyses described above were conducted for each dataset independently. See File S1 for details on the analyzed genomes.

Genomes were aligned by using the algorithm L-INS-I implemented in Mafft 7.471 [18]. After alignment, datasets of 29,712 (BA.5.2.3), 29,751 (BM.1.1.1), and 29,754 (XBF) bp long were produced. Manual check and editing were performed by means of the software Unipro UGENE v.35 [19]. The software jModeltest 2.1.1 [20] was utilized to identify the best probabilistic model for genome evolution through a maximum likelihood optimized search. Bayesian Inference (BI) was employed to estimate the times of the most recent common ancestor and evolutionary rate. This was accomplished using the software BEAST 1.10.4 [21] with runs consisting of 200 million generations under various demographic and clock models. The selection of the best model was determined through the Bayes Factor test [22], comparing the 2lnBF values of the marginal likelihoods as described by Mugosa et al. [9]. Additionally, BEAST was employed to draw the Bayesian Skyline Plots (BSP) and lineage thorough times for three subsets (BA.5.2.3, BA.2.75.3, and XBF). These analyses involved 200 million generation runs under the Bayesian Skyline Model with the uncorrelated log-normal relaxed clock model. For BSP analyses, all available genomes were included for each analyzed lineage (see File S1). Each genome included in the analysis possessed high quality, high coverage, and complete sampling date information.

All datasets used in this study were obtained by downloading genomes from the GISAID portal repository (https://gisaid.org/ accessed on 4 May 2023). For details on the used genomes and Authorship see File S1. 

The Breakpoint of the recombination event was inspected using a dataset consisting of all the genomes investigated from the two parental lineages and the recombinant lineage (BA.5.2.3 + BA.2.75.3 + XBF).

The mutations that define the analyzed SARS-CoV-2 lineages were identified by using consensus sequences obtained with a cutoff of 75% sequence prevalence among all available sequences. This cutoff value was chosen in alignment with the threshold utilized by the GISAID lineage comparison tool (https://gisaid.org/lineage-comparison/, accessed on 4 May 2023). Once identified, the mutations were confirmed by comparing the results with the “Lineage Comparison” web page provided by GISAID [17].

### 2.2. Structural Analyses 

Homology models of BA.2.75.3, BA.5.2.3, and XBF Spike RBDs (Receptor binding domain) and NTDs (N-terminal domain) were calculated with the Modeller 10.3 [23]. The PDB structures that were used as templates are denoted by codes 6M0J and 7B62 for RBD and NTD, respectively. Model structures were displayed and analyzed with the graphic program PyMOL (Schrodinger LLC, New York, NY, USA, 2015) [24]. FoldX 5.0 was applied to optimize the side chain conformation of the obtained models using the function “RepairPDB” [25]. To sample fluctuations of side chain conformations and interactions, 100 alternative homology models of RBD and NTD domains were built by Modeler. Indeed, the Modeler refinement stage of homology modelling produces alternative models differing in conformational details, such as side chain rotamers. Side chain conformations of each model were optimized in terms of energy and non-bonding interactions using the FoldX 5.0 “RepairPDB” function. For all models, structural properties were assessed to determine their average values and standard errors. Net charges were predicted using PROPKA3 [26], with a reference pH of 7.0. Surface electrostatic potential was calculated using the APBS program [27] and visualized as a two-dimensional projection using the SURFMAP software [28]. SURFMAP utilizes a “molecular cartography” approach to project a protein’s three-dimensional surface onto a two-dimensional plane. This allows for the analysis and comparison of the distribution of different physicochemical features over the protein surface.

Additionally, model structures of complexes between ACE2 and RBDs of the three variants BA.2.75.3, BA.5.2.3, and XBF were built with Modeler using the complex reported in PDB structure 6M0J as a template. 

The interaction energies between Spike RBDs and ACE2 were predicted using different methods. The Foldx 5.0 suite, specifically the “AnalyseComplex” tool, was employed to calculate the interaction energies. Foldx 5.0 utilizes an empirical force field that considers various free energy terms such as electrostatic interactions, hydrogen bonds, desolvation, and van der Waals contacts. Additionally, the MM/GBSA (Molecular Mechanics/Generalized Born Surface Area) method available in the HawkDock server [29] and PRODIGY [30] were utilized. MM/GBSA HawkDock combines molecular mechanics calculations with continuum solvation methods to compute binding free energies for macromolecules. PRODIGY, on the other hand, predicts binding affinities by analyzing the interfacial contacts between subunits. These approaches provide insights into the binding strengths and affinities between Spike RBDs and ACE2 through the calculation of interaction energies and interfacial contacts.

## 3. Results

### 3.1. Phylodynamics

**Figure 1:**
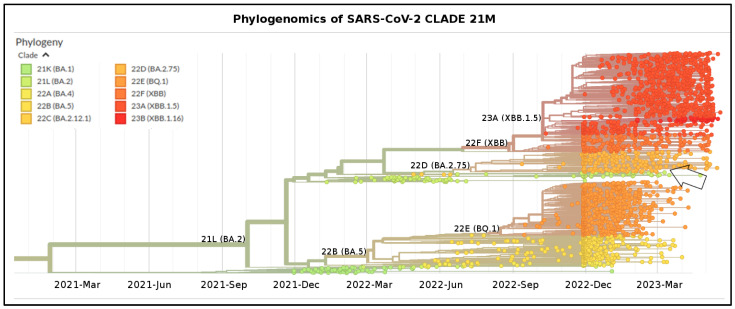
Highlight of the Omicron Clade (GISAID Clade 21M) in the time-scaled phylogenetic tree of a representative global subsample of 2350 of 2827 SARS-CoV-2 genomes sampled between November 2021 and May 2023. Arrow indicates genomes of XBF. Figure has been edited by using the software GIMP 2.8 (available at https://www.gimp.org/downloads/oldstable/, accessed on 24 May 2023). See Table 1 for details on Nextstrain clade, Pango lineage, and WHO labels. WHO, World Health Organization, Geneva, Switzerland.

Phylogenomic reconstruction (Figure 1) indicates that XBF genomes clustered within the not-monophyletic GISAID Clade 21L. More specifically, and as expected, they are evolutionarily close to genomes of BA.2 which represent their ancient progenitor. The results of the Bayes Factor analysis on the four datasets indicated that the Bayesian Skyline Model, implemented under the lognormal uncorrelated relaxed clock model, provided a significantly better fit to the data compared to other tested demographic and clock models (2lnBF = 16.4). The Time of the Most Recent Common Ancestor (TMRCA) of the clade composed by XBF is placed 167 days before 20 April 2023, i.e., 4 November 2022, with a date interval confidence of 182–151 days (i.e., 20 October 2022–20 November 2022) and its generation occurred between middle June and early November 2022.

**Figure 2:**
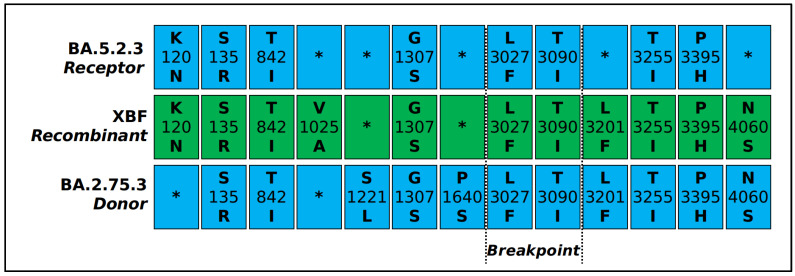
Scheme showing the viral genome characterizing mutation of the recombinant XBF and parental lineages BA.5.2.3 and BA.2.75.3. Dotted lines indicate the putative region of the recombination event (breakpoint). * indicates the lack of the mutation in that site.

The breakpoint has been individuated between the nucleotide positions 5186 and 9865 in the SARS-CoV-2 reference genome NC_045512.2. This region corresponds to the second half of the ORF1a gene (see Figure 2). 

**Figure 3:**
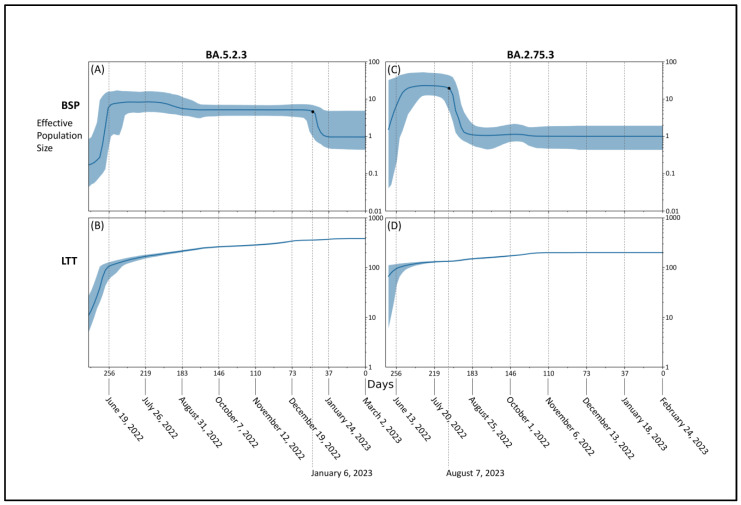
Bayesian skyline plot and lineages through time of SARS-CoV-2 BA.5.2.3 (**A**,**B**) and BA.2.75.3 (**C**,**D**) lineages. The viral effective population size (**A**,**C**) and the number of lineages (**B**,**D**) in the *y*-axis are shown as a function of days (*x*-axis).

Bayesian Skyline Plot (BSP) of the parental lineage BA.5.2.3 (Figure 3A) showed that viral population size reached the peak about 256 days before 2 March 2023 (i.e., 19 June 2022), starting its plateau phase with a substantial flattened genetic variability lasting for about 200 days, i.e., 6 January 2023, when the viral population size decreased drastically. Lineages through times plot (Figure 3B) confirmed this trend with a smoother curve of the lineages growing starting from about 256 days before 2 March 2023 (i.e., 19 June 2022).

BSP of the parental lineage BA.2.75.3 (Figure 3C) indicated that after the initial expansion in population size, the peak was reached around 256 days before 24 February 2023 (i.e., 13 June 2022) when the first plateau started, which lasted for about 55 days, i.e., 7 August 2022, when genetic variability and population size drastically decreased starting its last plateau that is still lasting. Lineages through times plot (Figure 3D) indicate that the maximum number of lineages has been achieved around 256 days before 24 February 2023, i.e., 13 June 2022.

**Figure 4:**
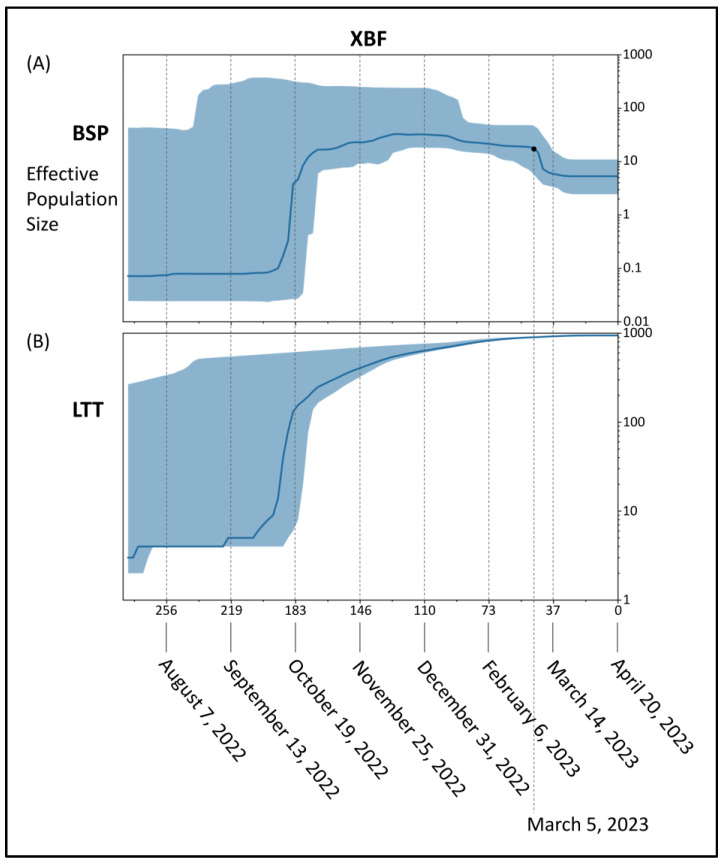
Bayesian skyline plot (**A**) and lineages through time (**B**) of the recombinant lineage SARS-CoV-2 XBF. The viral effective population size (**A**) and the number of lineages (**B**) in the *y*-axis are shown as a function of days (*x*-axis).

BSP of the recombinant XBF (Figure 4A) showed an initial period characterized by a flattened genetic variability, after which an increase of the viral population size occurred with a very severe curve peaking about 183 days before 20 April 2023 (i.e., 19 October 2022). After that genetic variability suffered slight oscillations until about 46 days before 20 April 2023 (i.e., 5 March 2023) when the population size reduced for the last time starting its last plateau that is still lasting. Lineages through times plot (Figure 4B) indicate a consistent increase in the number of lineages started about 183 days before 20 April 2023 (i.e., 19 October 2022) and then a slight increase with a very mild curve. 

The evolutionary rate of the four tested lineages amount to 4.949 × 10^−4^ [95% HPD 4.1988 × 10^−4^–6.0175 × 10^−4^], 8.1170 × 10^−4^ [95% HPD 6.2913 × 10^−4^–1.0031 × 10^−3^] and 4.8480 × 10^−4^ [95% HPD 2.1901 × 10^−4^–6.5616 × 10^−4^] subs/sites/years for BA.5.2.3, BA.2.75.3 and XBF, respectively. 

### 3.2. Structure

**Figure 5:**
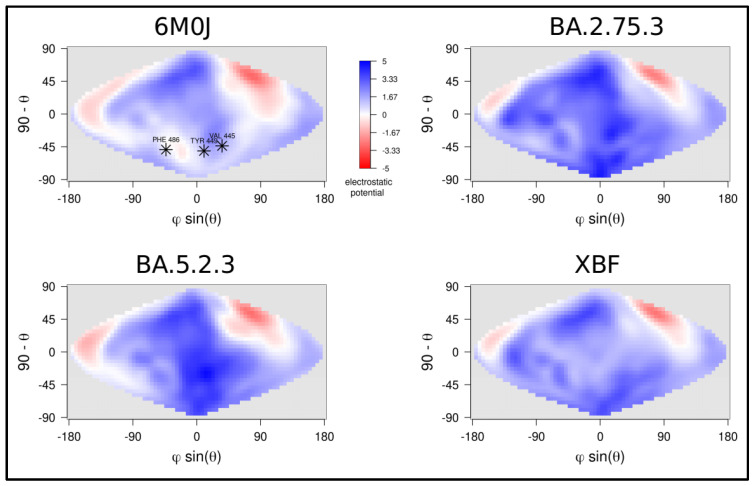
Comparison of surface electrostatic potential projections of RBDs of variants BA.2.75.2, BA.5.2.3, and XBF. The projection for the Wuhan RBD (6M0J) is reported as a reference. Asterisks in these projections mark the position of the RBD interface to ACE2. The color scale is reported beside the Wuhan projections. Numerical values are expressed as kT/e units.

Structural evolution of SARS-CoV-2 Spike from Wuhan strain to Omicron has proceeded through the fine-tuning of Spike physicochemical properties. In this work, some physicochemical properties of three related variants have been compared. The net charge has been calculated with PROPKA for each of the three NTDs and RBDs as a quantitative measure of differences among the surface electrostatic surfaces (Table 2). Apparently, BA.2.75.3 has a more positively charged RBD while BA.5.2.3 has a more positive NTD domain. Distributions of the RBD surface charge of the three variants are shown in Figure 5. 

Surface electrostatic potential distribution appears to be similar in the three domains, particularly in correspondence with the Receptor Binding Motif. The impact of the mutation at the interface ACE2-RBD has been assessed by prediction of the interaction energy by three independent methods: FoldX5, PRODIGY, and MM/GBSA (Table 3). Overall, BA.2.75.3 seems to have a more stable interaction with ACE2 compared with the other two variants.

**Figure 6:**
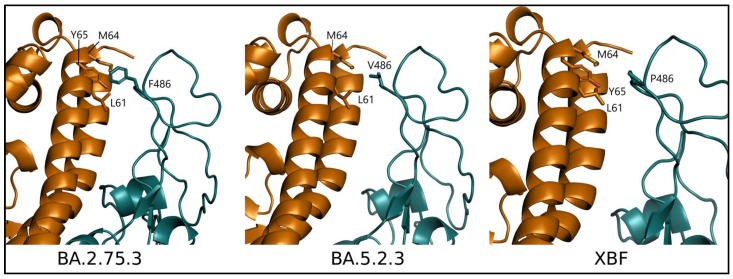
Comparison of variant complexes between ACE2 (orange) and RBD (deep teal) represented as cartoon models. The side chains of RBD position 486 are displayed along with ACE2 interacting residues as labeled stick models.

Among the three lineages considered, XBF shows three unique mutations within RBD: R346T, F486P, and F490S. Only F486P belongs to the interface to ACE2, and the same mutation occurs in variant XBB.1.5 (Figure S1). As observed in this case, the replacement of Phe with Pro decreases the local backbone flexibility [31] and removes in part the hydrophobic interactions with the ACE2 residues L61, M64, and Y65 (Figure 6). 

In the same position, BA.5.2.3 shows the mutation F486V that occur also in variants BA.4, BA.5, and BQ.1 [32]. The presence of Val in position 486 maintains only in part the hydrophobic interactions occurring between original Phe and ACE2 residues (Figure 6). Accordingly, interaction energy predicted for BA.5.2.3 tends to be slightly lower than XBF in two out of three predictions (Table 3) while BA.2.75.3 shows the lowest interaction energy among the three variants.

BA.2.75.3 and XBF NTDs are identical to variant CH.1.1. while BA.5.2.3 NTD is identical to variant BQ.1 [32]. A comparison of their electrostatic surface projections (Figure S2) confirms that BA.5.2.3 NTD has an overall more positive charge and shows how the potential distribution is altered in correspondence with deletion 69–70.

## 4. Discussion

Recombination represents the process through which the genetic material is exchanged between two separate organisms, resulting in the creation of an “offspring” that possesses a unique combination of traits that are not present in either parental lineage [33].

The SARS-CoV-2 XBF recombinant represents the latest outcome of a recombination event that took place during the ongoing COVID-19 pandemic. Like all newly identified variants, XBF requires a rigorous assessment of its genomic distinctions from its parental lineages to determine its real potential for spreading and contagiousness, as well as its pathogenic characteristics, including immune evasion. In this study, we aimed to gain a comprehensive understanding of the evolutionary and structural patterns of the SARS-CoV-2 recombinant XBF by means of a genome-based approach that utilized all available genomes in GISAID as of 4 May 2023. Phylogenomic reconstruction indicates that the genomes of XBF cluster together in a monophyletic group, which, in turn, falls within the wide and heterogeneous GISAID Clade 21L (BA.2). More specifically, the XBF clade is placed in the basal position of GSAID Clade 22D (BA.2.75). This is not surprising considering that one of the two parental lineages is a descendant of BA.2.75, i.e., BA.2.75.3. In the recombination event, the parental lineage BA.2.75.3 acted as the donor, while BA.5.2.3 served as the *Acceptor*, following the terminology of Focosi and Maggi [10], which defines the *Donor* and *Acceptor* as the strains represented in greater and lesser amounts, respectively [10]. Accordingly, the spike mutation profiles of XBF and BA.2.75.3 are very similar, differing only by three mutations. The confirmation of the parental lineages’ roles in the recombination event is reinforced by the location of the breakpoint, which occurs in the SARS-CoV-2 reference genome (NC_045512.2) between nucleotide positions 5186 and 9865, corresponding to the second half of the ORF1a gene. Within the gene, this range corresponds to amino acid positions 1640–3201. Accordingly, most of the mutations in XBF are shared with BA.2.75.3. In fact, XBF has the same genomic makeup as BA.5.2.3 up to P1640S, and from L3201F onwards, it has the genomic makeup of BA.2.75.3. The mutation P1640S (nucleotide position 4918..4920) is the last one inherited from BA.5.2.3, where proline was replaced by serine with a mutation in the first codon position (CCT-TCT). Similarly, the first mutation inherited from BA.2.75.3 is L3201F (nucleotide position 4918..4920), where leucine was replaced by phenylalanine, also with a mutation in the first codon position (CTT-TTT). The breakpoint of the recombination event is located in the region between these two mutations. Because this region is highly conserved, it is impossible to identify the exact point more accurately. This region contains only two amino acid mutations (L3027F and T3090I) that are common to both parental lineages and XBF.

Although recombination odds are not linked to the evolutionary path, it is interesting to note the occurrence of a recombination event between different lineages and ancestors (BA.5 and BA.2). The common ancestor to all analyzed genomes of XBF is estimated to have existed 167 days before 4 May 2023 (the most recent collection date), which is 4 November 2022. The time-calibrated tree suggests that it was likely generated between mid-June and early November 2022. This molecular dating calibration aligns with the first detected genome of XBF (EPI_ISL_16557356), which was isolated in Austria on 27 July 2022, from a 75-year-old male. During that period, the parental lineages BA.5.2.3 and BA.2.75.3 were prevalent in Asia, with a genome lineage prevalence of 41% and 48%, respectively. Recombination events typically occur when there is a high prevalence of parental lineages, which tend to experience a surge in contagion before recombining. Thus, it can be speculated that the recombination events occurred in Asia, where the lineage initially expanded and reached a genome lineage prevalence of 66% by late November [11]. Following the initial expansion, XBF became prevalent in Oceania, where it reached a genome lineage prevalence of 90% by mid-February 2023. Currently, it is not as common in both Oceania and Asia, but it has become widespread in Europe, with a relatively high genome lineage prevalence.

Independently of the area of XBF diffusion, phylogenomic reconstruction did not show evidence of great expansion capability. Indeed, the recombinant XBF and its direct sublineages exhibit an evolutionary pattern characteristic of an evolutionary dead-end lineage with no further epidemiologically relevant descendants that present features of concern. The branch’s length before the clade of XBF denotes the lack of rapid diversification, and in the current reconstruction, it does not highlight any features typical of an epidemiologically dangerous lineage at the beginning of its evolutionary path. Similar conditions have been observed in recent variants and lineages that raised several concerns in 2022 and 2023. Indeed, BA.2.75, BQ.1, XBB, XBB.1.5, BF.7, and CH.1.1 at the beginning of their life and evolutionary path caused many concerns, but molecular in-depth analysis proved their poor demographic expansion capability and low odds of becoming dangerous in the near future [11,16,31,32,34,35,36], which eventually happened. Less recently, a similar condition was observed in the BA.2.12.1 variant, which has not generated any new sub-lineages and has experienced a decline in its global genomic sequence prevalence over time, until its almost complete disappearance. As of today, the recombinant lineage XBF appears to be common in Europe, but according to the proposed dating results, XBF circulated undisturbed in Asia for several months before the surge in cases and the growth of genomic prevalence. As recently pointed out for the last investigated variants, this is not a feature of a highly expansive variant [11,16,31,32,34,35,36], which typically spreads much faster in terms of the number of infections and population size, as was the case with the Omicron variant (B.1.1.529), for instance, which became predominant in a short period [37]. The Bayesian Skyline Plot (BSP) analysis reveals an initial period of approximately 80 days with a flattened genetic variability and a small viral population size for XBF. As depicted in the lineage through time plot, XBF exhibited an increase in genetic variability and population size starting around 19 October 2022. There was a rapid growth phase with a steep curve, reaching a peak within about 15 days, followed by a plateau phase from 19 October 2022 onwards. This period of rapid growth provided XBF with an evolutionary advantage, allowing it to outcompete the parental lineages BA.5.2.3 and BA.2.75.3 in Asia, similar to what occurred with the recombinant XBB lineage [11]. However, as of the present time, XBF has not expanded globally, and around 10 November 2022, its population size declined due to decreased genetic variability. During this period, there was a kind of vicariance between XBF and its parental lineages, possibly because the variant lacked sufficient strength to prevail over all others. BA.5.2.3 and BA.2.75.3 never became dominant, and their genome sequence prevalence was already low even before the emergence of XBF. Interestingly, the evolutionary rates of BA.5.2.3 and BA.2.75.3, estimated at 4.9 × 10^−4^ and 8.1 × 10^−4^ subs/site/year, respectively, were not particularly fast, as confirmed by the BSP plots. The peak phase for both variants occurred around mid-June 2022, with a modest level of lineage increase. This growth arrest in their population size allowed for an increase in the genomic sequence prevalence of XBF, despite its similar low evolutionary rate of 4.8 × 10^−4^ substitutions per site per year. It is worth noting that the evolutionary rate of XBF is significantly lower, especially when compared to the initial lineage of SARS-CoV-2 (Wuhan-Hu-1 variant), which had an evolutionary rate of approximately 6.58 × 10^−3^ subs/site/year [38]. This makes the evolutionary rate of XBF approximately a factor of 10^−1^ slower than Wuhan-Hu-1.

A comparison of a selection of structural properties of NTDs and RBDs of the three variants suggests that no significant difference can be detected among them. Based on this, it could be speculated that the convergent evolution of SARS-CoV-2, and positive selection in response to immune pressure has brought to such an optimized state of some virus structural and functional properties that they cannot be further improved in terms of stability or receptor affinity while immunoescaping abilities are strongly promoted. In fact, a vast literature suggests that the newly emerging variants possess various degrees of immune evasion potential (see i.a., Uraki et al. [39], Qu et al. [40], and Khurade et al. [41]). Supposedly, the evolution of the latest SARS-CoV-2 variants is mainly driven by the acquisition of new immune evasion abilities. In this context, it is quite common to detect subtle differences in the distribution of electrostatic potential over the surfaces of the variants’ NTDs and RBDs, which may influence interactions with antibodies or molecular cell components that are relevant for infection and transmissibility.

It should be noted that the study reported here is based on theory and all on models, and not directly on experimental results. However, the usefulness of models for interpreting experimental observations or designing new experiments is widely accepted.

The structural analysis of the analyzed SARS-CoV-2 lineages reveals that the mutations primarily occur in the NTD (N-terminal domain), RBD (receptor-binding domain), and RBM (receptor-binding motif) regions, while the S2 region remains highly conserved. This observation is consistent with the fact that the NTD, RBD, and RBM regions of the spike protein contain numerous potent B cell epitopes that can effectively trigger a robust neutralizing antibody response. However, there is no evidence regarding the high fitness of XBF. Overall, it can be speculated that variations in genomic composition are the result of genetic drift, which allows the virus to constantly adapt to its host, but it is not necessarily linked to a competitive advantage.

## 5. Conclusions

In conclusion, the genomic survey of the SARS-CoV-2 recombinant XBF lineage suggests that while it initially displayed a rapid expansion, causing concerns, there is currently no evidence of high expansion capabilities or a contagion rate higher than other ongoing or previously circulating lineages. Recombination is a common evolutionary mechanism in the Coronaviridae family and RNA viruses in general. XBF is the second recombinant lineage (after XBB) that has shown growth worth investigating, becoming regionally dominant. However, its expansion capability appears to be limited, with a peak reached in mid-October 2022 and a subsequent decrease in genetic variability and population size since mid-March. It is currently in a plateau phase. The data suggest an initial rapid growth followed by a sustained period of limited genetic variability, which is far from the expansion capabilities of an epidemiologically dangerous lineage, as observed at the beginning of the pandemic when the population size exhibited an extremely steep curve (see, e.g., Lai et al. [42]). 

Ongoing genome-based surveillance is crucial for all SARS-CoV-2 lineages and variants to detect any potential new expansions. The structural interpretation of mutations helps in formulating hypotheses to explain and predict the epidemiological behavior of variants. Future mutations may make XBF more concerning or give rise to new subvariants. Therefore, attention should be focused on its descendants to assess their expansion capability and biological features for a better understanding of the pandemic.

## Figures and Tables

**Table 1:** Nextstrain clade, Pango lineage and WHO labels of the investigate lineages showed in Figure 1.

Nextstrain Clade	Pango Lineage	WHO Label
21L (Omicron)	BA.2	o (Omicron)
22A (Omicron)	BA.4	o (Omicron)
22B (Omicron)	BA.5	o (Omicron)
22C (Omicron)	BA.2.12.1	o (Omicron)
22D (Omicron)	BA.2.75	o (Omicron)
22E (Omicron)	BQ.1	o (Omicron)
21K (Omicron)	BA.1	o (Omicron)
23A (Omicron)	XBB.1.5	o (Omicron)
23B (Omicron)	XBB.1.6	o (Omicron)
22F (Omicron)	XBB	o (Omicron)

**Table 2:** Net charge of NTDs and RBDs for XBF, BA.2.75.3, and BA.5.2.3.

Lineage	RBD	NTD
XBF	5.43 ± 0.01	0.12 ± 0.03
BA.2.75.3	6.47 ± 0.01	0.12 ± 0.03
BA.5.2.3	5.22 ± 0.01	0.92 ± 0.03

**Table 3:** Predicted interaction energy between ACE2 and variant RBDs expressed in Kcal/mol.

Method	XBF	BA.2.75.3	BA.5.2.3
FoldX 5.0	−4.29 ± 0.30	−5.40 ± 0.36	−4.68 ± 0.31
PRODIGY	−10.97 ± 0.04	−11.12 ± 0.04	−11.11 ± 0.04
MM/GBSA	−63.72 ± 0.77	−68.23 ± 0.74	−63.09 ± 0.63

## Data Availability

Genomes analyzed in the present study were taken from GSAID database and are available at https://gisaid.org/, accessed on 4 May 2023.

## Supplementary Materials

The following supporting information can be downloaded at: https://www.mdpi.com/article/10.3390/microorganisms11071824/s1, Figure S1: Position of the three unique XBF mutations in RBD domain. Residues are represented as labelled stick models. N- and C-terminal sides are indicated by the letters N or C, respectively. Orange arrow marks the ACE2 interface. Figure S2: Comparison of surface electrostatic potential projections of NTDs of variants BA.2.75.2, BA.5.2.3, and XBF. BA.2.75.2 and XBF NTDs are identical and are represented by a single projections. Asterisks in S projections BA.2.75.2/XBF mark the N-terminal side (Gln1) and the deletion 69–70 (His53, Val54). The color scale is reported beside this projection. Numerical values are expressed as kT/e units. Table S1: Characterizing mutations of the compared lineages. Mutations labeled in red font are mutations of interest (MOI). File S1: Acknowledgement and reference of Authors who deposited analyzed genomes in GISAID portal.
