# Supplementary material for: SARS-CoV-2 Recombinants: Genomic Comparison between XBF and Its Parental Lineages
Source: Microorganisms. 2023 Jul 17;11(7):1824. doi: 10.3390/microorganisms11071824 (PMC10383834; doi:10.3390/microorganisms11071824)
Supplement: Supplementary file 1 [file microorganisms-11-01824-s001.zip › File_S1.pdf]

SUPPLEMENTAL TABLE

Data Availability

GISAID Identifier:	EPI_SET_230429cw
doi:	10.55876/gis8.230429cw

All genome sequences and associated metadata in this dataset are published in GISAID's EpiCoV database. To view the contributors of each individual sequence with details such as accession number, Virus name, Collection date, Originating Lab and Submitting Lab and the list of Authors, visit 10.55876/gis8.230429cw

Data Snapshot

- EPI_SET_230429cw is composed of 234 individual genome sequences.
- The collection dates range from 2022-06-02 to 2022-12-13;
- Data were collected in 15 countries and territories;
- All sequences in this dataset are compared relative to hCoV-19/Wuhan/WIV04/2019 (WIV04), the official reference sequence employed by GISAID (EPI_ISL_402124). Learn more at https://gisaid.org/WIV04.

SUPPLEMENTAL TABLE

Data Availability

GISAID Identifier:	EPI_SET_230429fx
doi:	10.55876/gis8.230429fx

All genome sequences and associated metadata in this dataset are published in GISAID's EpiCoV database. To view the contributors of each individual sequence with details such as accession number, Virus name, Collection date, Originating Lab and Submitting Lab and the list of Authors, visit 10.55876/gis8.230429fx

Data Snapshot

- EPI_SET_230429fx is composed of 50 individual genome sequences.
- The collection dates range from 2022-12-16 to 2022-12-27;
- Data were collected in 7 countries and territories;
- All sequences in this dataset are compared relative to hCoV-19/Wuhan/WIV04/2019 (WIV04), the official reference sequence employed by GISAID (EPI_ISL_402124). Learn more at https://gisaid.org/WIV04.

SUPPLEMENTAL TABLE

Data Availability

GISAID Identifier: EPI_SET_230427dt
doi: 10.55876/gis8.230427dt

All genome sequences and associated metadata in this dataset are published in GISAID's EpiCoV database. To view the contributors of each individual sequence with details such as accession number, Virus name, Collection date, Originating Lab and Submitting Lab and the list of Authors, visit 10.55876/gis8.230427dt

Data Snapshot

- EPI_SET_230427dt is composed of 50 individual genome sequences.
- The collection dates range from 2022-12-27 to 2023-01-12;
- Data were collected in 14 countries and territories;
- All sequences in this dataset are compared relative to hCoV-19/Wuhan/WIV04/2019 (WIV04), the official reference sequence employed by GISAID (EPI_ISL_402124). Learn more at https://gisaid.org/WIV04.

SUPPLEMENTAL TABLE

Data Availability

GISAID Identifier: EPI_SET_230427nr
doi: 10.55876/gis8.230427nr

All genome sequences and associated metadata in this dataset are published in GISAID's EpiCoV database. To view the contributors of each individual sequence with details such as accession number, Virus name, Collection date, Originating Lab and Submitting Lab and the list of Authors, visit 10.55876/gis8.230427nr

Data Snapshot

- EPI_SET_230427nr is composed of 50 individual genome sequences.
- The collection dates range from 2023-01-12 to 2023-03-02;
- Data were collected in 5 countries and territories;
- All sequences in this dataset are compared relative to hCoV-19/Wuhan/WIV04/2019 (WIV04), the official reference sequence employed by GISAID (EPI_ISL_402124). Learn more at https://gisaid.org/WIV04.

SUPPLEMENTAL TABLE

Data Availability

GISAID Identifier:	EPI_SET_230429zn
doi:	10.55876/gis8.230429zn

All genome sequences and associated metadata in this dataset are published in GISAID's EpiCoV database. To view the contributors of each individual sequence with details such as accession number, Virus name, Collection date, Originating Lab and Submitting Lab and the list of Authors, visit 10.55876/gis8.230429zn

Data Snapshot

- EPI_SET_230429zn is composed of 151 individual genome sequences.
- The collection dates range from 2022-06-25 to 2022-12-05;
- Data were collected in 23 countries and territories;
- All sequences in this dataset are compared relative to hCoV-19/Wuhan/WIV04/2019 (WIV04), the official reference sequence employed by GISAID (EPI_ISL_402124). Learn more at https://gisaid.org/WIV04.

SUPPLEMENTAL TABLE

Data Availability

GISAID Identifier:	EPI_SET_230429yx
doi:	10.55876/gis8.230429yx

All genome sequences and associated metadata in this dataset are published in GISAID's EpiCoV database. To view the contributors of each individual sequence with details such as accession number, Virus name, Collection date, Originating Lab and Submitting Lab and the list of Authors, visit 10.55876/gis8.230429yx

Data Snapshot

- EPI_SET_230429yx is composed of 50 individual genome sequences.
- The collection dates range from 2022-10-07 to 2023-02-24;
- Data were collected in 12 countries and territories;
- All sequences in this dataset are compared relative to hCoV-19/Wuhan/WIV04/2019 (WIV04), the official reference sequence employed by GISAID (EPI_ISL_402124). Learn more at https://gisaid.org/WIV04.

SUPPLEMENTAL TABLE

Data Availability

GISAID Identifier:	EPI_SET_230429gx
doi:	10.55876/gis8.230429gx

All genome sequences and associated metadata in this dataset are published in GISAID's EpiCoV database. To view the contributors of each individual sequence with details such as accession number, Virus name, Collection date, Originating Lab and Submitting Lab and the list of Authors, visit 10.55876/gis8.230429gx

Data Snapshot

- EPI_SET_230429gx is composed of 1,867 individual genome sequences.
- The collection dates range from 2022-07-27 to 2023-04-20;
- Data were collected in 29 countries and territories;
- All sequences in this dataset are compared relative to hCoV-19/Wuhan/WIV04/2019 (WIV04), the official reference sequence employed by GISAID (EPI_ISL_402124). Learn more at https://gisaid.org/WIV04.
