# Supplementary material for: SARS-CoV-2 Recombinants: Genomic Comparison between XBF and Its Parental Lineages
Source: Microorganisms. 2023 Jul 17;11(7):1824. doi: 10.3390/microorganisms11071824 (PMC10383834; doi:10.3390/microorganisms11071824)
Supplement: Supplementary file 1 [file microorganisms-11-01824-s001.zip › Table_S1.pdf]

Table S1. Characterizing mutations of the compared lineages.

	**BA.2.75.3**	**BA.5.2.3**	**XBF**
**ORF1a**		K120N	K120N
	S135R	S135R	S135R
	T842I	T842I	T842I
			V1025A
	S1221L		
	G1307S	G1307S	G1307S
	P1640S		
	L3027F	L3027F	L3027F
	T3090I	T3090I	T3090I
	L3201F		L3201F
	T3255I	T3255I	T3255I
	P3395H	P3395H	P3395H
	DEL3675/3677	DEL3675/3677	DEL3675/3677
	N4060S		N4060S
**ORF1b**	P314L	P314L	P314L
	G662S		G662S
		T1050N	
	R1315C	R1315C	R1315C
	I1566V	I1566V	I1566V
	V1706I		V1706I
	T2163I	T2163I	T2163I
	T19I	T19I	T19I
	L24S	L24S	L24S
	DEL25/27	DEL25/27	DEL25/27
		DEL69/70	
	G142D	G142D	G142D
	K147E		K147E
	W152R		W152R
	F157L		F157L
	I210V		I210V
	V213G	V213G	V213G

S	G257S		G257S
	G339H		G339H
		G339D	
			R346T
	S371F	S371F	S371F
	S373P	S373P	S373P
	S375F	S375F	S375F
	T376A	T376A	T376A
	D405N	D405N	D405N
	R408S	R408S	R408S
	K417N	K417N	K417N
	N440K	N440K	N440K
	G446S		G446S
		L452R	
	N460K		N460K
	S477N	S477N	S477N
	T478K	T478K	T478K
	E484A	E484A	E484A
		F486V	
			F486P
			F490S
	Q498R	Q498R	Q498R
	N501Y	N501Y	N501Y
	Y505H	Y505H	Y505H
	D614G	D614G	D614G
	H655Y	H655Y	H655Y
	N679K	N679K	N679K
	P681H	P681H	P681H
	N764K	N764K	N764K
	D796Y	D796Y	D796Y
	Q954H	Q954H	Q954H
	N969K	N969K	N969K
**ORF3a**	T223I	T223I	T223I
**E**	T9I	T9I	T9I
	T11A		T11A

**M**		D3N	
	Q19E	Q19E	Q19E
	A63T	A63T	A63T
**ORF6**	D61L		D61L
**ORF8**	S84L	S84L	S84L
**N**	P13L	P13L	P13L
	DEL31/33	DEL31/33	DEL31/33
	R203K	R203K	R203K
	G204R	G204R	G204R
	S413R	S413R	S413R

The contents in red indicate Mutations of Interest.
