# Supplementary figures and images for: SARS-CoV-2 Recombinants: Genomic Comparison between XBF and Its Parental Lineages
Source: Microorganisms. 2023 Jul 17;11(7):1824. doi: 10.3390/microorganisms11071824 (PMC10383834; doi:10.3390/microorganisms11071824)

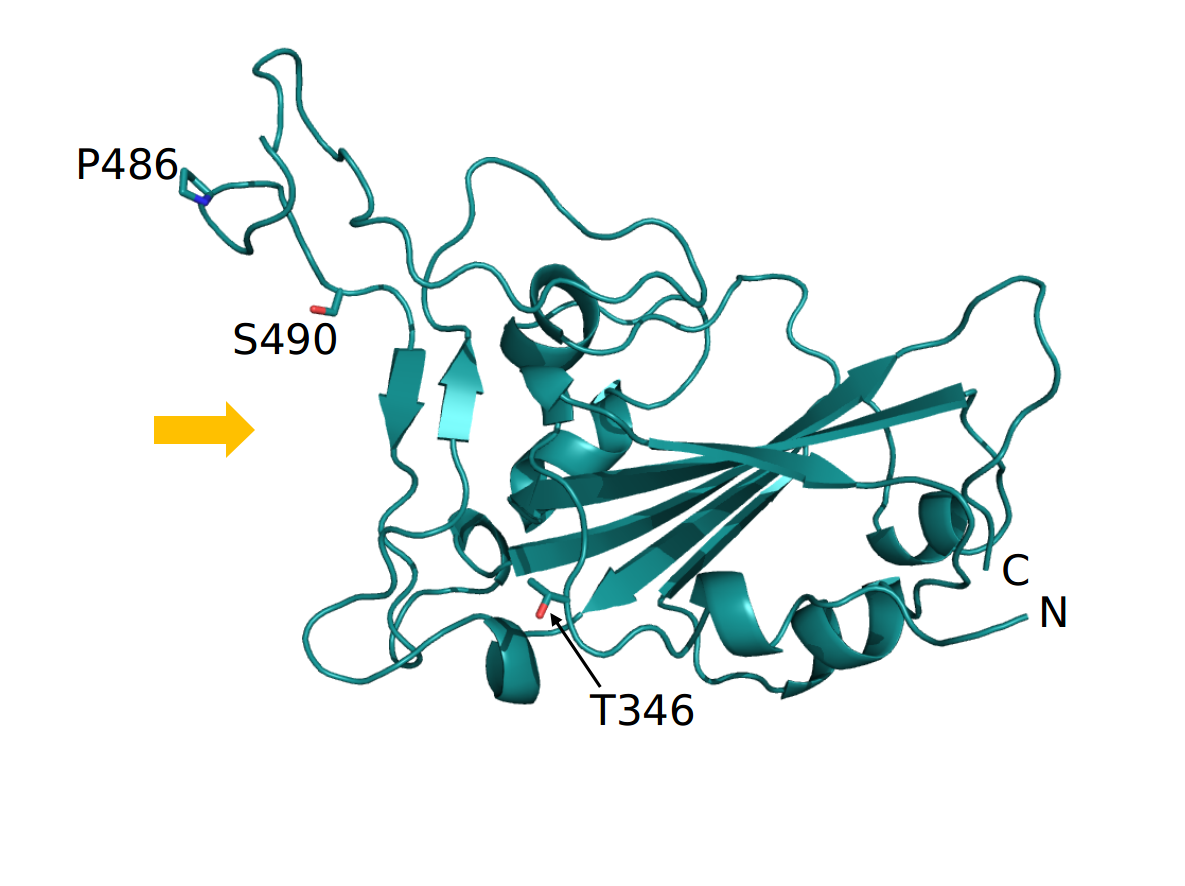

Supplement: Supplementary file 1 [file microorganisms-11-01824-s001.zip › Figure_S1.tif]

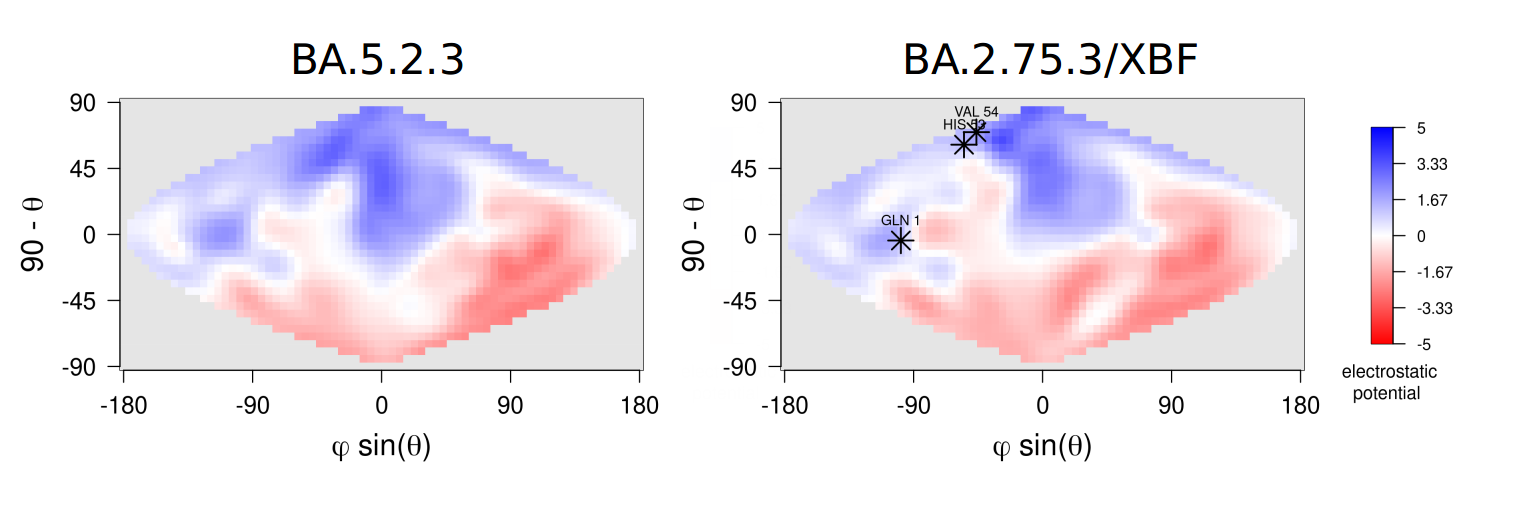

Supplement: Supplementary file 1 [file microorganisms-11-01824-s001.zip › Figure_S2.tif]
